# Heat Shock Lipolysis: Radiofrequency Combined with Cryolipolysis for the Reduction of Localized Subcutaneous Fat

**DOI:** 10.1155/2020/4093907

**Published:** 2020-02-07

**Authors:** S. Abboud, J. P. Hachem

**Affiliations:** ^1^Hôpital Erasme, Université Libre de Bruxelles, Route de Lennik 808, 1070 Bruxelles, Belgium; ^2^Centre Hospitalier Emile Mayrisch, L4240 Esch-sur-Alzette, Luxembourg

## Abstract

*Introduction*. Noninvasive procedures such as cryolipolysis, noncontact selective radiofrequency (RF), and laser selective fat heating have been shown to be safe and effective for the reduction of localized subcutaneous fat. *Material and Methods*. In this retrospective study, we describe the safety and efficacy of combining RF with cryolipolysis for localized unwanted fat after one single session. 69 patients, 61 females, and 8 males for a total of 75 treatments were included in this study. All patients underwent RF prior to and following cryolipolysis. Pictures (*n* = 24), taken before and after treatment, were used to clinically assess the physician G**lobal Aesthetic Improvement Scale (PhGAIS)**. In parallel, patients were asked to subjectively evaluate the efficacy of the treatment using the same scale (PaGIAS).

**Results:**

PhGIAS showed an improvement in 18 patients (73.46%), 5 (22.44%) were unchanged, and 1 (4.08%) worsened their appearance after treatment. The mean PaGIAS scored as “good improvement.”

**Conclusion:**

In conclusion, combining RF with cryolipolysis in one single session is safe and effective.

## 1. Introduction

Current trends in body contouring have gained a great popularity over the past 10 years as the patient/consumer demand has grown quite exponentially [1, 2]. Liposuction surgical procedures for fat reduction offer great results but are compromised by high postsurgical risks as well as relatively important financial costs and mostly overlong associated downtime [3].

Still, the current demand has led to the development of a number of noninvasive techniques, representing now the fastest growing area of aesthetic medicine [4]. These noninvasive technologies allow fast fat reduction with minimal pain and significant visible signs of improvement with virtually no downtime. Cold/freezing therapies have pioneered the market by the introduction of Coolsculpting, an FDA-approved technology for fat reduction since 2009 [5].

However, adipocytes are not only reactive to cold temperatures but also to acute heat shock [6, 7]. Since lipid-to-gel phase transition has been considered as a potential apoptotic signal for fat cell apoptosis following cryolipolysis [8], pre- and postlipocryolysis hyperthermal conditioning showed a significant increase in rat adipocyte destruction [9].

Hence, pre- and postheating technology applied to lipocryolysis also called “contrast lipocryolysis,” was evaluated in 10 subjects and showed significant fat reductions [10].

More recently, a noncontact selective radiofrequency (RF) device (Vanquish system, BTL) has been clinically proven to significantly reduce abdominal fat in 4 to 6 weekly sessions. The efficacy of this technique has been scientifically documented and offers a great alternative to cryolipolisis [11]. All the same, a 1060 nm diode laser was used to reach hyperthermic temperatures within fat tissue and was found to be effective and noninvasive for fat reduction in patients [12]. Alternatively, deep bulk heating of the skin by using 1064 nm Nd : YAG can be used in a super-long (PIANO® mode, Fotona) pulse modality. This seconds-long duration of the pulse allows sufficient heat diffusion to the underlying dermal fat without potentially injuring the epidermis [13, 14].

In this paper, we aimed to illustrate the efficacy of combining either noncontact selective RF with conventional cryolipolysis in one single treatment. We demonstrate the fast and significant decrease in unwanted fat as a result of “heat shock lipolysis”.

## 2. Materials and Methods

### 2.1. Patient Selection

Both male (*n* = 8) and female (*n* = 72) patients were included in this retrospective study with a mean age of 47 (range 23–72). Patients' exclusion criteria included history of cold urticaria and cryoglobulinemia. All patients were naïve to body remodeling therapies and signed an informed consent for picture publication. Pictures were taken immediately before and 6 weeks following the treatment in 24 patients. One single treatment session was performed per selected-to-treat area of interest totalizing the number of sessions to 75.

### 2.2. Cryolipolysis

All subjects received one treatment cycle on each area of interest (Table 1) using the Cooltech cryolipolysis device (Cocoon medical, Spain). The treatment consisted of either −8°C, 60 minute cooling cycle delivered with the standard parallel cooling plate vacuum applicator for the lower abdominal area (*n* = 8) or a −8°C, 60 minute cooling cycle delivered using a curved vacuum applicator for the flanks (*n* = 2), or a flat vacuum applicator at −7°C, 60 minute cooling cycle for the saddlebags (*n* = 2).

### 2.3. Noncontact Selective Radiofrequency

RF technology deployed by Vanquish (BTL Aesthetics, Czech Republic) uses oscillating electrical current forcing collisions between charged molecules and ions, which are then transformed into heat. Since fat biophysical characteristics behave like an insulator capable of polarization, it absorbs the high RF-related heat release from the RF applicator driving specific fat necrosis and consequent lipolysis. Patients lay underneath the device while the focused-field radiofrequency heats up the underlying. RF was performed 15 minutes prior and following cryolipolysis in 6 patients.

### 2.4. Patient's Safety Evaluation

Safety was evaluated by the incidence and duration of local and systemic AEs. Patients answered a questionnaire in the follow-up visit at the clinic, and any AEs noted by the patients were recorded.

### 2.5. Patient's Effectiveness and Satisfaction Evaluation

The effectiveness was evaluated on a subset of patients that were treated between the period of January 2016 and February 2018. 24 patients with pictures prior to and after treatment were scored by Physicians using the **Global Aesthetic Improvement Scale (Ph**GAIS; Table 1), while patients' GAIS (PaGIAS, *n* = 69; Table 2) was used to assess patients' self-satisfaction in the form of a questionnaire.

## 3. Results

The aim of this retrospective study was to evaluate the efficacy and the safety of combining both RF and cryolipolysis simultaneously in/and after one single session for the reduction of unwanted body fat. 75 treatments were performed on either the abdominal area (*n* = 60) and/or the flanks (*n* = 15) in 69 patients (61 females and 8 males).

**Figure 1:**
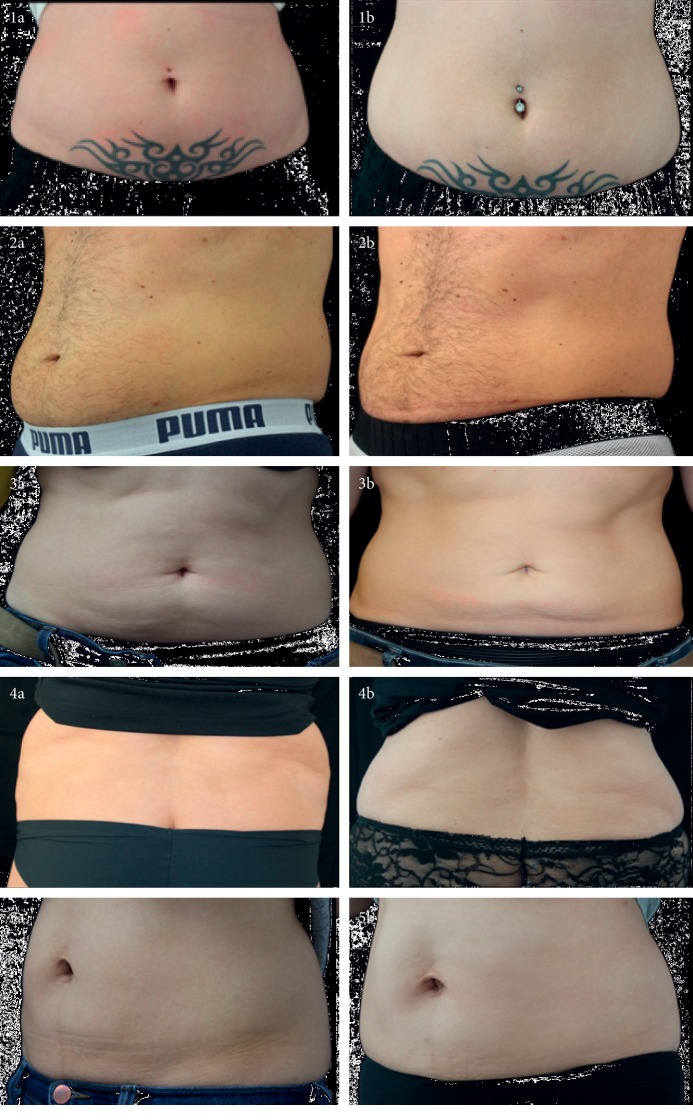
RF-assisted cryolipolysis accelerates the removal of fat excess. Either lower abdominal (1, 2, 3, and 5) or bilateral flanks (4); unwanted fat excess were treated by combining RF and cryolipolysis. Pictures were taken immediately before (a) as well as 6 weeks (b) after on single treatment session. A significant clinical difference could be observed as a consequence of RF-assisted cryolipolysis.

**Figure 2:**
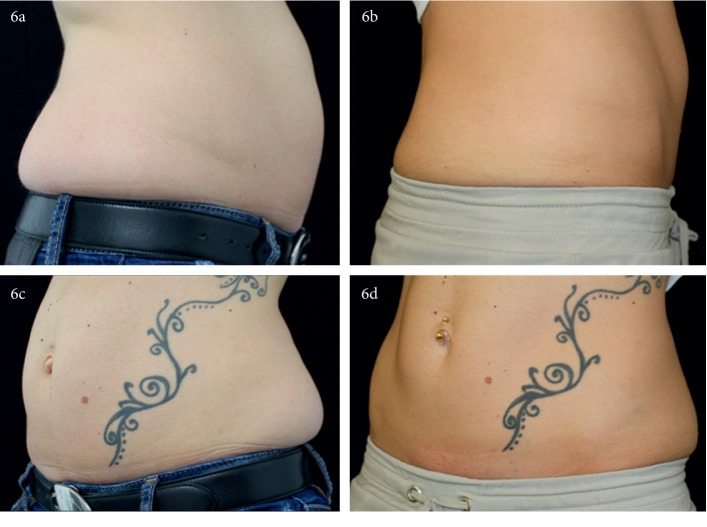
Multiple area treatment with RF-assisted cryolipolysis. Both lower abdominal fat excess (day 1) and bilateral flanks (day 2) in the same patient by combining RF and cryolipolysis. Pictures were taken immediately before (a) as well as 6 weeks (b) after one single treatment session. A significant clinical difference could be observed as a consequence of RF-assisted cryolipolysis.

RF was performed two times for 15 minutes each in a “sandwich” regimen before and after cryolipolysis. The effectiveness of such treatment was evaluated on a subset of patients (*n* = 24 patients) for whom both pre- and posttreatment photos were available. In these 24 patients, GAIS was scored by physicians (PhGIAS) by comparing posttreatment outcomes with baseline pictures; PhGIAS showed an improvement in 18 patients (73.46%), 5 (22.44%) were unchanged, and 1 (4.08%) worsened their appearance after treatment. Patients' GIAS (PaGIAS) is a self-evaluation process of patients (*n* = 69) scoring their treatment outcome by responding to a questionnaire. As illustrated in Figure 1, a significant reduction is observed in both lower fat abdominal (Figure 1, patients #1a, 2a, 3a, and 5a versus patients #1b, 2b, 3b, and 5b) and the flank area (Figure 1, patients #4a and 4b). All the same, when both lower abdomen and flanks were treated in a 24 hour interval period and further evaluated 6 weeks after treatment, a significant improvement was observed in all treated zones (Figure 2 patient #6 a vs. b).

In parallel, on a scale from “worse” to “very much improved” (Table 1), the patients were asked scores and were assessed as follows: worse = 0, no change = 1, improved = 2, much improved = 3 and very much improved = 4. The mean score of PaGIAS was 2.16.

## 4. Discussion

In this paper, we describe a new method for noninvasive lipolysis, where both RF and cryolipolysis are combined in one single session creating the conditions for “heat shock lipolysis.” 69 patients were treated by combining RF prior to and following cryolipolysis for localized unwanted fat in one single session treatment protocol. Both physician and patients' evaluation proved the safety and the efficacy of the method.

The efficacy of RF as a noninvasive technique to reduce subcutaneous fat in thighs and buttocks has been proven in a large body of research [4, 15–21]. In the majority of these studies, the patients received more than two sessions (2 to 16) over several weeks [4, 15–21].

The combination of pre- and postheating with cryolipolysis has already proven its effectiveness when compared with conventional lipolysis. Indeed, Pinto et al. showed in their study a 42.45% improvement in fat reduction by using contrast lipolysis [8, 10]. This phenomenon has also been observed by Pinto et al. on in vitro adipocytes models. These authors applied a hot preconditionning before cooling down the cells and finally warming them up again. They witnessed a higher rate of crystal formation and adipocyte destruction [9]. Similarly, we show that combining RF and cryolipolysis improved patients' outcome after one single session.

Many sessions are usually required in order to obtain a satisfying result using cryotherapy; Stevens et al. repeated four times the treatment while Ferraro et al. needed three to four cycles of cryotherapy [22, 23]. In our study, a single session of cryotherapy is sufficient as to reduce the fat excess significantly, providing more comfort to the patient.

Many studies have shown the usual complications following cryolipolysis, such as erythema, bruising, swelling, sensitivity, and pain [2, 4, 5, 21, 22]. Meanwhile, in a wide study of 528 patients who underwent cryotherapy, Stevens et al. reported only 3 cases of mild to moderate pain/neuralgia [23]. Other studies have also shown some adverse effects due to radiofrequency, such as erythema and pain [4]. Katz and Doherty. showed no major complications but still a mild to moderate tenderness in some patients after a single session of radiofrequency [24]. Even though these side effects might resolve within weeks, they remain a source of discomfort for the patient. In our study, no serious adverse events were reported except some pain in 10 patients that resolved spontaneously after 1 week.

## 5. Conclusion

Combining RF prior to and following cryolipolysis in one single session is a simple, safe, and efficient method for the treatment of unwanted fat.

## Figures and Tables

**Table 1:** Physician Global Aesthetic Improvement Scale (PhGAIS).

Very much improved	Optimal cosmetic result from the initial condition
Much improved	Marked improvement from the initial condition
Improved	Obvious improvement from the initial condition
No change	The appearance is identical to the original condition
Worse	The appearance is worse than the original condition

**Table 2:** Patient Global Aesthetic Improvement Scale (PaGAIS).

Very much improved	4
Much improved	3
Improved	2
No change	1
Worse	0
